# Sensor-Based Dynamic Response and Stress Estimation in Monolithic and Laminated Glass Beams Under Soft Impact Loading

**DOI:** 10.3390/s26185929

**Published:** 2026-09-19

**Authors:** Natalia García-Fernández, F. Pelayo, Manuel Aenlle, Mario López-Gallego, María Jesús Lamela-Rey

**Affiliations:** Department of Construction and Manufacturing Engineering, University of Oviedo, Campus de Gijón, Zona Oeste, Edificio 7, 33203 Gijón, Spainmjesuslr@uniovi.es (M.J.L.-R.)

**Keywords:** laminated glass, soft impact loading, accelerometers, sensor-based stress estimation, operational modal analysis, structural assessment

## Abstract

Monolithic and laminated glass elements are increasingly used in structural applications, where their response to impact loading must be assessed to ensure safety. For soft impacts, simplified simulations based only on the initial impact velocity may be inaccurate because the transient coupling between the impactor and the glass depends on the impactor stiffness and mass. This study investigates the dynamic response of monolithic and laminated glass beams under soft impact loading using accelerometers, an instrumented impact hammer, strain gauges, operational modal analysis, and simplified finite element models. The influence of three impactor heads and three hammer masses is examined. Two approaches are assessed: a modal simulation driven by the measured impact-force history and a sensor-based method that reconstructs stresses from measured structural responses and experimentally identified mode shapes. Comparisons with experimental measurements yield approximate absolute-peak errors of 2.1% for the monolithic-beam acceleration and 1.5% for the laminated-beam stress, normalised by the maximum absolute measured response in each case, with corresponding Pearson correlation coefficients of 0.972 and 0.943. The results show that accounting for the measured impact-force history and combining response measurements at discrete locations with modal information can provide efficient estimates of dynamic response and stress without explicitly modelling the impactor-glass contact.

## 1. Introduction

Recent architectural trends have considerably increased the use of monolithic and laminated glass products in structural applications, including floor glazing, facades, security glass and blast- or ballistic-resistant glazing. These elements must be designed and assessed to satisfy the applicable safety requirements.

Laminated glass is a layered material composed of two or more monolithic glass plies bonded by one or more polymeric interlayers under pressure and temperature in an autoclave. Polymeric interlayers exhibit viscoelastic behaviour; therefore, their mechanical properties depend on time and temperature [1,2]. Polyvinyl butyral (PVB) is the most commonly used interlayer. In analytical and numerical models, glass is generally represented as a linear-elastic material, whereas PVB is commonly represented as linear viscoelastic [3,4,5]. Recent experiments on processed interlayers, including PVB, further demonstrate the combined influence of temperature and strain rate on their mechanical response [6].

The mechanical behaviour of glass elements subjected to dynamic loading, particularly impact, must be verified according to the applicable standards [7,8,9]. The European standard EN 12600:2003 [7] addresses the behaviour of monolithic and laminated glass panels subjected to pendulum impacts representative of human impact. Numerous experimental programmes have followed this standard to characterise glass behaviour and to calibrate numerical models used to predict displacements and stresses under impact loading [10,11,12,13,14,15]. Recent investigations have characterised the force histories generated by soft-body impact on glass curtain walls [16] and assessed laminated glass in intact and post-fracture states using an EN 12600-based procedure [17].

The EN 12600 test [7] uses a pendulum frame and a soft impactor consisting of two pneumatic tyres. When the test is simulated numerically, an energy-balance approach is commonly used to estimate the impactor velocity, which is then prescribed as the initial velocity. Other simplified procedures calibrate the impact velocity or the response through an equivalent pressure load. However, these procedures often neglect the transient coupling between the impactor and the glass and the nonlinear behaviour of the tyre system. In addition, human impacts may differ depending on the body region involved, which motivates the investigation of different impact conditions. Measurements on full-scale curtain walls also show that the force-time input is sensitive to the impactor type and impact configuration [16].

A finite element analysis may explicitly model the complete impactor-glass system to reproduce the interaction more realistically [10,13]. However, such models are computationally demanding and difficult to implement. For laminated glass, the analysis is further complicated by the time- and temperature-dependent viscoelastic response of the interlayer [3,4,5].

Recent finite element studies have investigated hard-body impact and fracture in multilayer laminated safety glass [18], and the effects of layer thickness, impactor velocity, and interlayer stiffness under static and impact loading [19]. For soft-body pendulum tests, neural networks trained on numerical simulations have also been proposed to predict maximum principal stresses and deformations in flat and curved glass panels [20]. These studies illustrate current approaches to modelling impact response and reducing computational effort.

The effective-thickness concept has been proposed to estimate deflections and stresses in laminated glass beams through simplified monolithic models with time- and temperature-dependent equivalent properties [3,4,5,21,22]. Recent complementary approaches include experimental determination of effective thickness through stress comparisons with monolithic reference specimens [23], and identification of effective complex moduli by combining experimental modal analysis with finite element modelling of an automotive laminated-glass component [24].

In this study, two simplified approaches are investigated and experimentally assessed for the dynamic response and stress estimation of monolithic and laminated glass beams subjected to soft impact loading. The individual components of these approaches, including modal response reconstruction, mode-shape expansion, and stress-effective modulus formulations, are based on established methods and previous developments. The contribution of the present work lies in their combined application and experimental assessment under soft-impact conditions, including the influence of impactor stiffness and mass. The first approach combines experimentally identified modal parameters with the measured impact-force history and a numerical model, whereas the second combines sparse structural-response measurements, experimental modal information, mode-shape expansion, and a stress-effective modulus to reconstruct stress histories in laminated glass. Both approaches are assessed through selected comparisons with experimental measurements for impactors of different stiffnesses.

The contribution is the application and experimental assessment of this combined framework for soft-impact response and stress estimation, rather than a new modal-expansion or stress-effective-modulus formulation [21,25,26,27]. Using the measured force history or structural response incorporates the dynamics of the actual impact without requiring the release height as an input to these reconstructions. An initial velocity inferred from height alone does not specify the force-time history unless impactor–specimen interaction is also represented. The present validation is based on experimental measurements; a comparison of accuracy with alternative energy-based methods is beyond the scope of the present study.

Because the second approach estimates stress histories from measurements at discrete locations, it may support sensor-based structural assessment and provide input for future damage-detection and localisation studies when direct strain measurement at all locations of interest is not practical [28,29].

## 2. Soft Impact Tests and Sensor-Derived Impact Force

**Figure 1:**
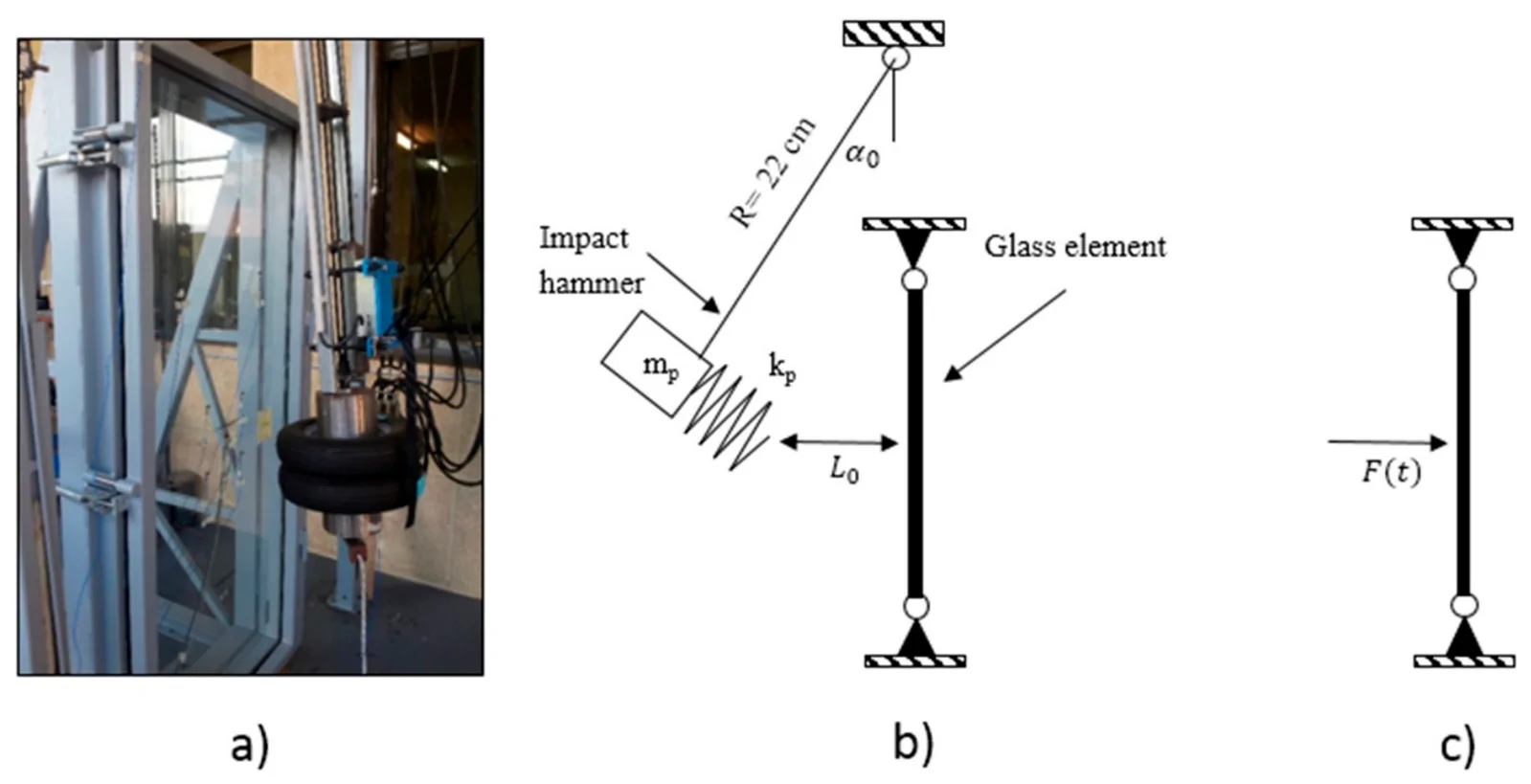
(**a**) Soft impactor according to EN 12600; (**b**) soft-impact model; and (**c**) model driven by the force-time history.

Impact tests involve an impactor that is released from a specified height and strikes the specimen (Figure 1). Different configurations can be used, including ballistic pendulums, Charpy tests, and drop towers. For soft impacts on glass elements, a standard pendulum device is commonly used [7].

For a soft impact, the initial velocity can be obtained from the release height. Nevertheless, the response also depends on the impactor stiffness, the impactor-to-specimen mass ratio, and the damping introduced by the contact system (Figure 1). The transient coupling between the impactor and the glass may therefore make the simulation computationally demanding and difficult to implement.

When transient coupling occurs, prescribing only the initial velocity may lead to inaccurate predictions because not all the impactor kinetic energy is immediately transferred to the glass. Part of the energy is temporarily stored or dissipated through deformation of the soft impactor.

The release height defines the test condition, but it is not required as an input when the measured impact force drives the model or when stress is reconstructed from the measured structural response. These measurements reflect the actual excitation and structural dynamics for that event. Agreement is assessed directly against measured acceleration and stress. No independent energy- or momentum-balance verification was performed; agreement in the response comparisons should not be interpreted as such a verification.

If only the glass specimen is represented in the finite element model, the measured force acting on the specimen should be applied instead of explicitly modelling the impactor [10]. This force depends on the impactor stiffness, hammer mass, and release height.

When a load cell cannot be used, the contact-force component may be estimated from the corresponding measured impactor acceleration using an appropriate effective inertial mass:

(1) $$F(t)=m_{p}a(t)$$

where $m_{p}$ denotes the effective inertial mass associated with the measured acceleration component. It equals the total impactor mass for pure translation when other force contributions in the measurement direction are negligible. For a rotating pendulum, the mass must account for rotational inertia and the locations of the accelerometer and contact point; the total hammer mass cannot automatically be used. Force and acceleration polarities must be chosen consistently.

A complementary comparison of measured hammer force and acceleration is presented in Appendix B. The pendulum geometry gives an approximate effective mass of 1.05 kg for this comparison. The main response calculations in this paper use the measured force histories.

## 3. Sensor-Based Stress Estimation from Structural Response

**Figure 2:**
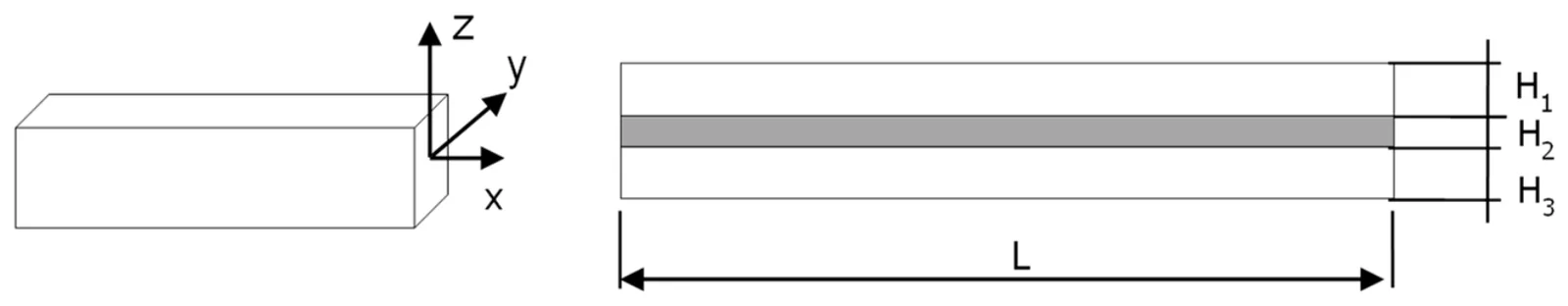
Monolithic and laminated glass beams.

Consider a planar beam bending in the x-z plane (Figure 2). The x-axis is oriented along the beam and the positive z-axis is directed upwards, as shown in Figure 2. The transverse displacement w(x,t) is defined as positive in the +z direction, tensile normal stresses are taken as positive, and $M_{y}$ denotes the bending-moment component about the positive y-axis.

Under the Euler-Bernoulli assumption, the bending moment and curvature are related by:

(2) $$-EI_{y}\frac{d^{2}w(x,t)}{dx^{2}}=M_{y}(x,t)$$

where E is the Young’s modulus, $I_{y}$ is the second moment of the cross section about the y-axis, w(x,t) is the vertical displacement (Figure 2), and $M_{y}$ is the bending moment. Accordingly, Navier’s law can be applied, and the stresses can be determined by:

(3) $$\sigma \left(x,t\right)=\frac{M_{y}(x,t)}{I_{y}}z$$

where z is the distance from the neutral axis to the point of interest in the section. Equations (2) and (3) can be combined; the following relation between stress and curvature is derived:

(4) $$\sigma \left(x,t\right)=-Ez\frac{d^{2}w(x,t)}{dx^{2}}$$

In structural dynamics, the response can be decomposed into modal contributions using the mode-superposition method [30]:

(5) $$w\left(x,t\right)=\sum_{i=1}^{Nmodes}\phi_{i}\left(x\right)q_{i}\left(t\right)$$

where $\phi_{i}\;(x)$ and $q_{i}(t,T)$ are the i−th mode shape and the i−th modal coordinate, respectively.

Substituting Equation (5) into (4) gives the stress as [25]:

(6) $$\sigma \left(x,t\right)=-\sum_{i=1}^{Nmodes}Ez\phi_{i}^{\prime \prime }\left(x\right)q_{i}\left(t\right)$$

where superscript ″ indicates the second derivative with respect to x and E is Young’s modulus. For a rectangular section, the largest absolute bending stresses occur at its upper and lower surfaces. The signed stress at the upper surface is:

(7) $$\sigma_{top}\left(x,t\right)=-\sum_{i=1}^{Nmodes}E\frac{H}{2}\phi_{i}^{\prime \prime }\left(x\right)q_{i}\left(t\right)$$

where H is the beam thickness. The lower-surface stress has the opposite sign, and the maximum absolute stress through the section is the absolute value of the upper-surface stress.

In laminated glass beams, polymeric interlayers are commonly modelled as linear-viscoelastic materials; consequently, their mechanical properties depend on time and temperature. For a beam (Figure 2) with two glass layers with thicknesses $H_{1}$ and $H_{3}$, respectively, and one interlayer with thickness $H_{2}$, the stress at the top of layer 1 can be obtained from the expression [26]:

(8) $$\sigma_{1}\left(x,t,T\right)≅-\frac{H_{1}}{2}\cdot \sum_{i=1}^{Nmodes}{E_{1\sigma i}\left(T\right)\cdot \phi }_{i}^{\prime \prime }\left(x\right)\cdot q_{i}\left(t,T\right)$$

Similarly, the stress at the bottom of layer 3 is obtained as:

(9) $$\sigma_{3}\left(x,t,T\right)≅\frac{H_{3}}{2}\cdot \sum_{i=1}^{Nmodes}E_{3\sigma i}\left(T\right)\cdot \phi_{i}^{\prime \prime }\left(x\right)\cdot q_{i}\left(t,T\right)$$

where $E_{1\sigma i}\;(T)$ and $E_{3\sigma i}\;(T)$ are constant time-domain stress-effective Young’s moduli, which depend on the geometry and mechanical properties of the glass and PVB [26]. These time-domain stress-effective Young’s moduli are obtained from:

(10) $$E_{1\sigma i}\left(T\right)=E_{1\sigma eff}\left(\omega_{i},T\right)$$

and

(11) $$E_{3\sigma i}\left(T\right)=E_{3\sigma eff}\left(\omega_{i},T\right)$$

where $\omega_{i}$ is the natural frequency of the i−th mode.

The analytical expressions for $E_{1\sigma eff}(\omega_{i},T)$ and $E_{3\sigma eff}(\omega_{i},T)$ are frequency-domain effective Young’s moduli. Their expressions were obtained by the authors in a previous paper [27], and have been included in Appendix A.

The transverse displacement and thickness coordinate are taken as positive upwards, and tensile stress is positive. The opposite signs in Equations (8) and (9), and in Equations (13) and (14), follow from this convention. The stress-effective moduli provide the corresponding magnitudes of the stress–curvature factors [21,26,27].

If the experimental modal parameters of the beam (natural frequencies, mode shapes, and damping ratios) are known from a previous modal analysis, and the experimental response time histories w(x,t) are measured at several points of the structure, the vector of experimental modal coordinates $q_{x}\;(t,T)$ can be estimated by:

(12) $$q_{x}\left(t,T\right)={\left[\phi_{x}\right]}^{-1}{\;w}_{x}\left(t,T\right)$$

where subscript ‘x’ indicates experimental data, ${\left[\phi_{x}\;\left(x\right)\right]}^{-1}$ represents the inverse or pseudoinverse of the experimental mode-shape matrix and $w_{x}\;(t,T)$ is the vector of experimental responses.

The response processing follows the modal stress-estimation framework described in [25]. The measured accelerations were converted to displacement by frequency-domain double integration [31,32]. Low-frequency components were suppressed before integration to limit the amplification of noise and drift near zero frequency. In the present beam tests, only a high-pass filter was used, with a cutoff below 5 Hz. The high-pass filter uses a smooth cosine transition, while a Hanning window is applied to each signal segment before Fourier transformation. After filtering and double integration at non-zero frequencies, the displacement signal is recovered by inverse Fourier transformation and addition of overlapping segments.

The high-pass cutoff is selected by examining the measured response or modal-coordinate spectra together with the identified modal frequencies. The aim is to suppress low-frequency components unrelated to structural vibration while keeping the filter transition below the first structural frequency of interest. The first measured frequencies of the beams were 31.013 Hz and 30.16 Hz for the monolithic and laminated beams, respectively, well above the cutoff used here. This frequency separation provides a physical basis for removing low-frequency noise while retaining the modal response used for stress reconstruction.

A separate manuscript currently under review examines the selection of the method parameters and the influence of their variation on reconstruction errors. The present study focuses on the application of the method to the reported soft-impact experiments.

If Equation (12) is substituted into Equations (8) and (9), the stress at the top of layer 1, at any longitudinal position x within the expanded model, can be obtained from [26]:

(13) $$\sigma_{1}\left(x,t,T\right)≅-\frac{H_{1}}{2}\cdot \sum_{i=1}^{Nmodes}E_{1\sigma i}\left(T\right)\cdot \phi_{xpi}^{\prime \prime }\cdot q_{xi}\left(t,T\right)$$

Similarly, the stress at the bottom of layer 3 is:

(14) $$\sigma_{3}\left(x,t,T\right)≅\frac{H_{3}}{2}\cdot \sum_{i=1}^{Nmodes}E_{3\sigma i}\left(T\right)\cdot \phi_{xpi}^{\prime \prime }\cdot q_{xi}\left(t,T\right)$$

where $\phi_{xpi}^{\prime \prime }$ are the second spatial derivatives of the experimental mode shapes expanded to the unmeasured DOFs using one of the techniques proposed in the literature [25,33].

In this study, mode shapes obtained from a monolithic finite element model were used for the expansion. Assuming that the experimental mode shapes can be expressed as a linear combination of the numerical mode shapes [34]:

(15) $$\left[\phi_{x}]=\left[\phi_{FE}\right]\cdot [T\right]$$

where subscripts ‘x’ and ‘FE’ indicate experimental and numerical mode shapes, respectively. The transformation matrix [T] can be estimated using the components corresponding to the measured DOFs:

(16) $$\left[T]={\left[\phi_{FE}^{m}\right]}^{-1}\;[\phi_{xm}\right]$$

where superscript ‘m’ in Equation (16) indicates measured DOFs. If there are fewer modes than measured DOFs, the pseudoinverse must be used in Equation (16).

Finally, the expanded experimental mode shapes are obtained from Equation (15).

## 4. Monolithic Glass Beam

### 4.1. Modal Analysis

A monolithic annealed glass beam with the mechanical and geometrical properties listed in Table 1 was tested. The beam was clamped at both ends between rubber pads in a standard glass impact frame [7].

**Figure 3:**
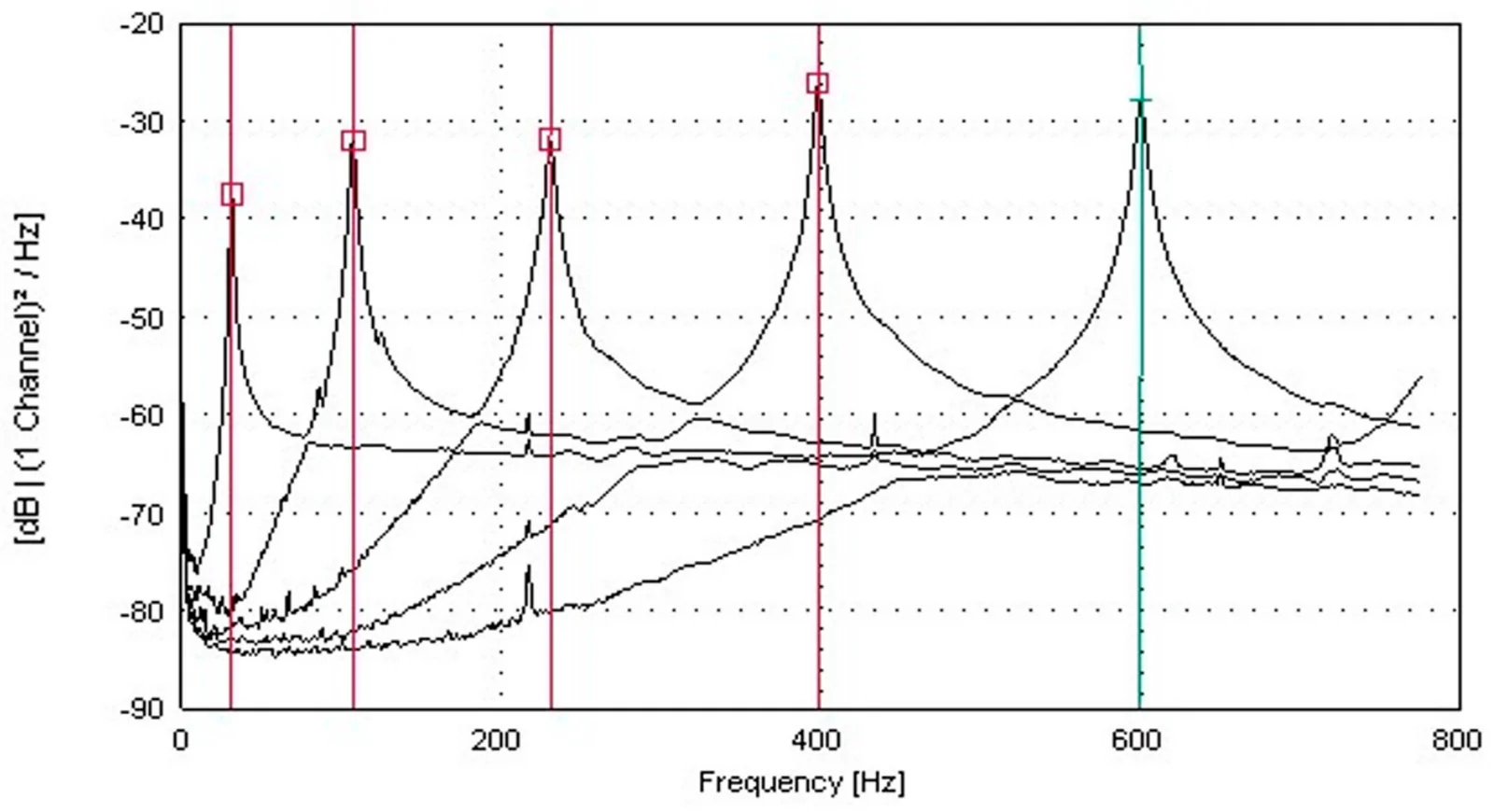
Singular-value decomposition of the response spectral-density matrix for the monolithic-beam OMA test.

The dynamic behaviour of the beam was characterised using operational modal analysis. Five PCB 352C15 accelerometers with a sensitivity of 10 mV/g were uniformly distributed along the beam. The signals were acquired with a National Instruments cDAQ system (NI 9188 chassis and NI 9234 module) at a sampling frequency of 2132 Hz, and the test was performed at 20 °C. The modal parameters were estimated in the frequency domain using the Enhanced Frequency Domain Decomposition (EFDD) technique [35,36,37] implemented in ARTeMIS Modal 8.0. Figure 3 presents the singular-value decomposition of the response spectral-density matrix, and Table 2 lists the first five natural frequencies and corresponding damping ratios.

The beam was excited with a small impact hammer by applying impacts at locations and instants selected randomly in space and time, a procedure suitable for laboratory OMA [36].

A numerical model was assembled in ABAQUS using quadrilateral shell elements with reduced integration (S4R). The glass was assigned a Young’s modulus of 70 GPa, a Poisson’s ratio of 0.22, and a mass density of 2500 kg/m^3^. The numerical natural frequencies and mode shapes are presented in Table 2. Across the frequency range considered, the discrepancies between the experimental and numerical natural frequencies were below 10%.

### 4.2. Impact Tests

**Figure 4:**
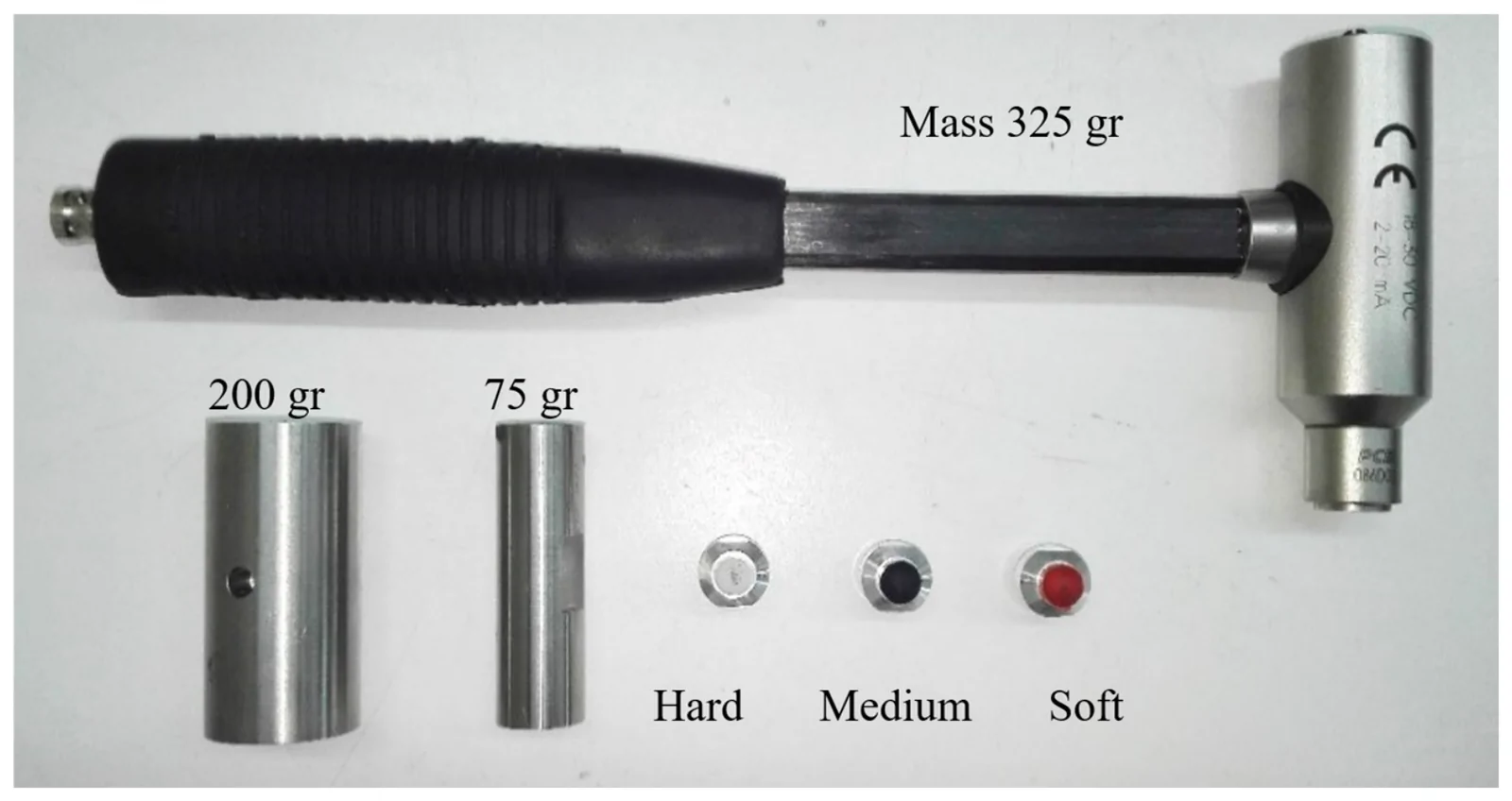
Impact hammer, hammer masses and impactor heads used in the experiments.

The beam was subjected to impact tests at its midpoint using a PCB 086D05 instrumented impact hammer with a sensitivity of 0.23 mV/N (Figure 4).

**Figure 5:**
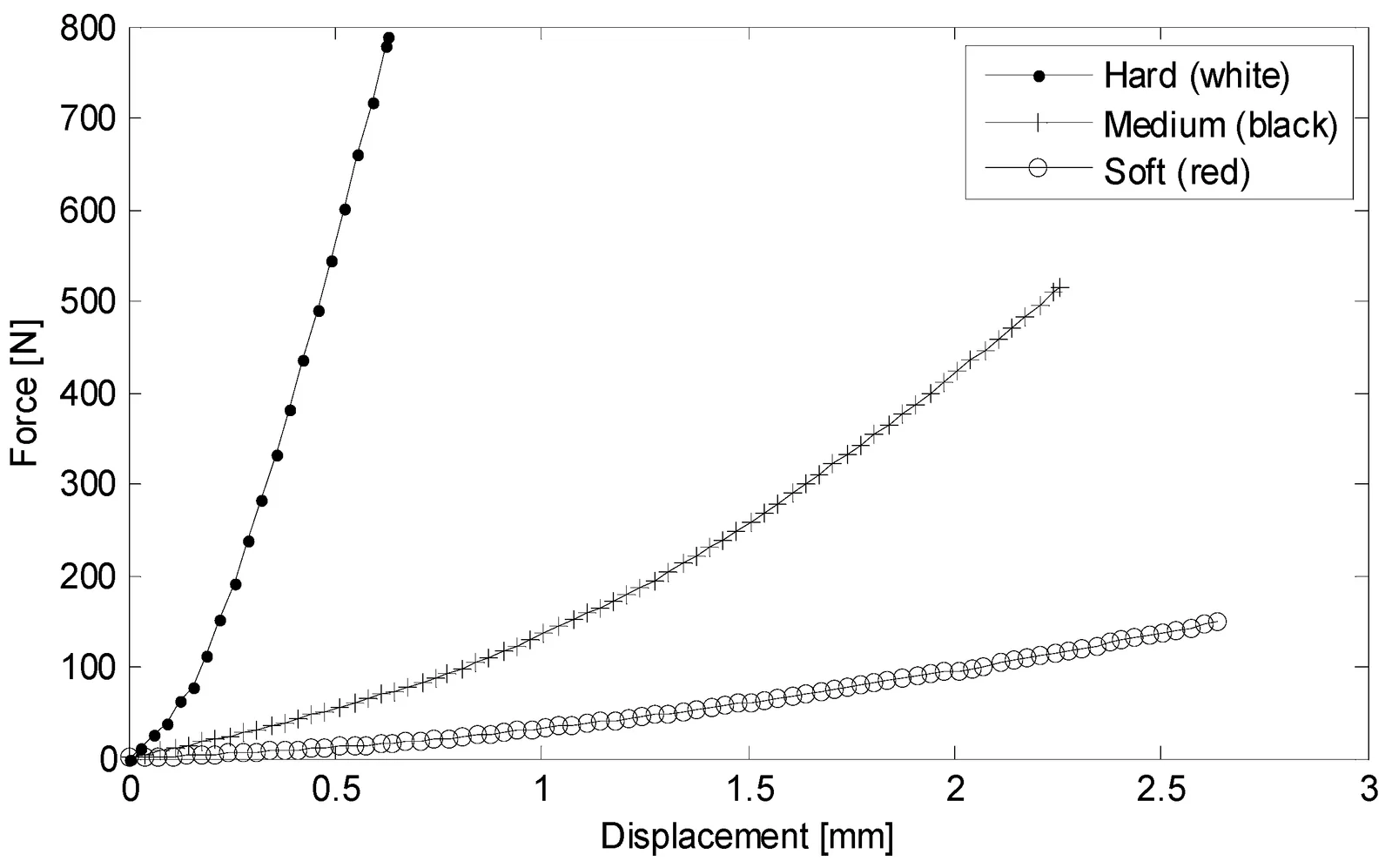
Force-displacement response of the three impactor heads.

The test configuration used for the modal tests was retained for the impact tests. The beam responses were sampled at 20,400 Hz. Three impactor heads and two additional hammer masses of 75 and 200 g were considered. The compressive force-displacement response of each impactor head was characterised experimentally through compression tests using an axial MTS testing machine with a maximum load of 5 kN. Figure 5 shows the measured force-displacement curves for the three heads. The terms soft, medium, and hard are used here as comparative labels for the measured compression responses in Figure 5, rather than as specified values of a constant spring stiffness. The nonlinear force-displacement curves do not define a unique stiffness independent of displacement, and no single stiffness value is used as an input to the modal simulations. The compression curves provide a comparative characterisation of the heads; equivalence to their effective dynamic stiffness during impact has not been established. The simulations in Section 4.4 instead use the measured impact-force histories, so they do not require conversion of these compression curves into a dynamic contact stiffness.

After the correct operation of the complete measurement chain had been verified, one impact was performed for each impactor-head and hammer-mass configuration. The curves therefore correspond to individual tests rather than averages. The objective was to estimate the structural response for each specific measured loading condition, rather than to assess statistical repeatability.

### 4.3. Experimental Results

This section presents the impact-test results obtained using the impact hammer shown in Figure 4 while varying the impactor head and hammer mass. The release distance was kept constant for all tests at $L_{0}=140$ mm (Figure 1).

#### 4.3.1. Influence of the Impactor Head

**Figure 6:**
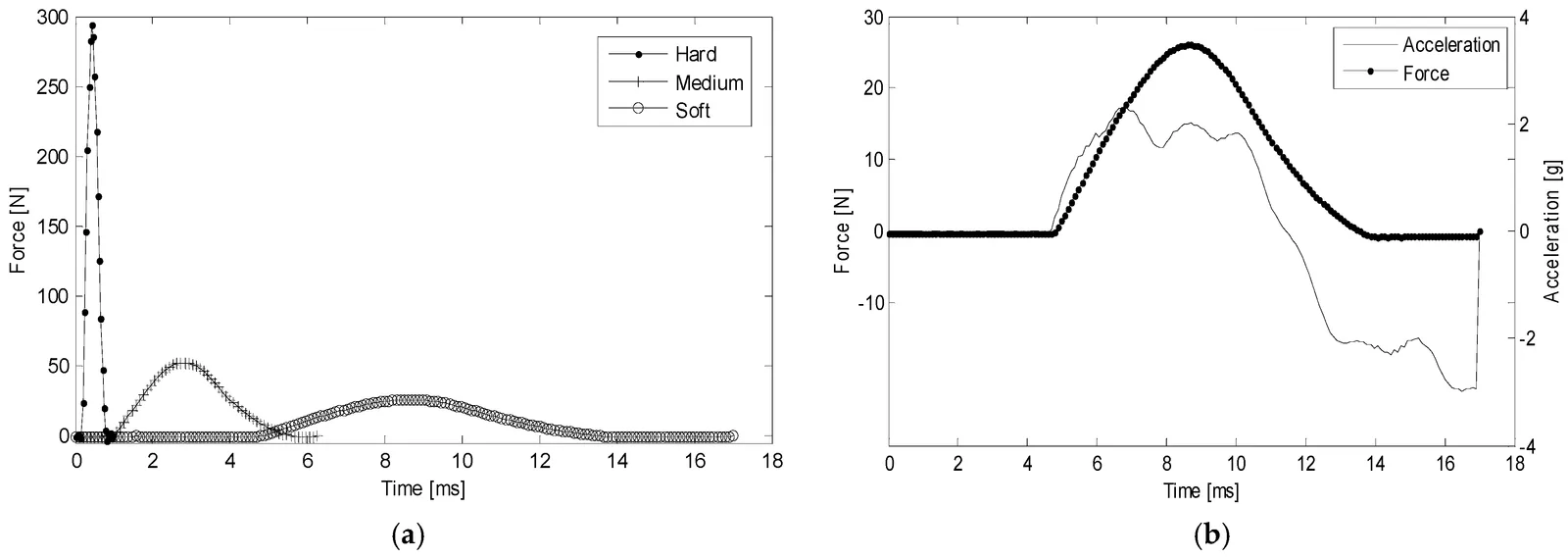
(**a**) Force-time histories for the three impactor heads; (**b**) impact force and acceleration measured at the beam midpoint.

Three impactor heads with different stiffnesses were used. Figure 6a presents the force-time histories measured with the hammer load cell.

Figure 6b illustrates the coupling between the impactor and the glass beam by comparing the impact force with the acceleration measured at the beam midpoint. During the transient coupling interval (approximately 4–14 ms), the beam first accelerates in the impact direction and then reverses its motion. Contact ends when the measured force returns to zero.

#### 4.3.2. Influence of Hammer Mass

**Figure 7:**
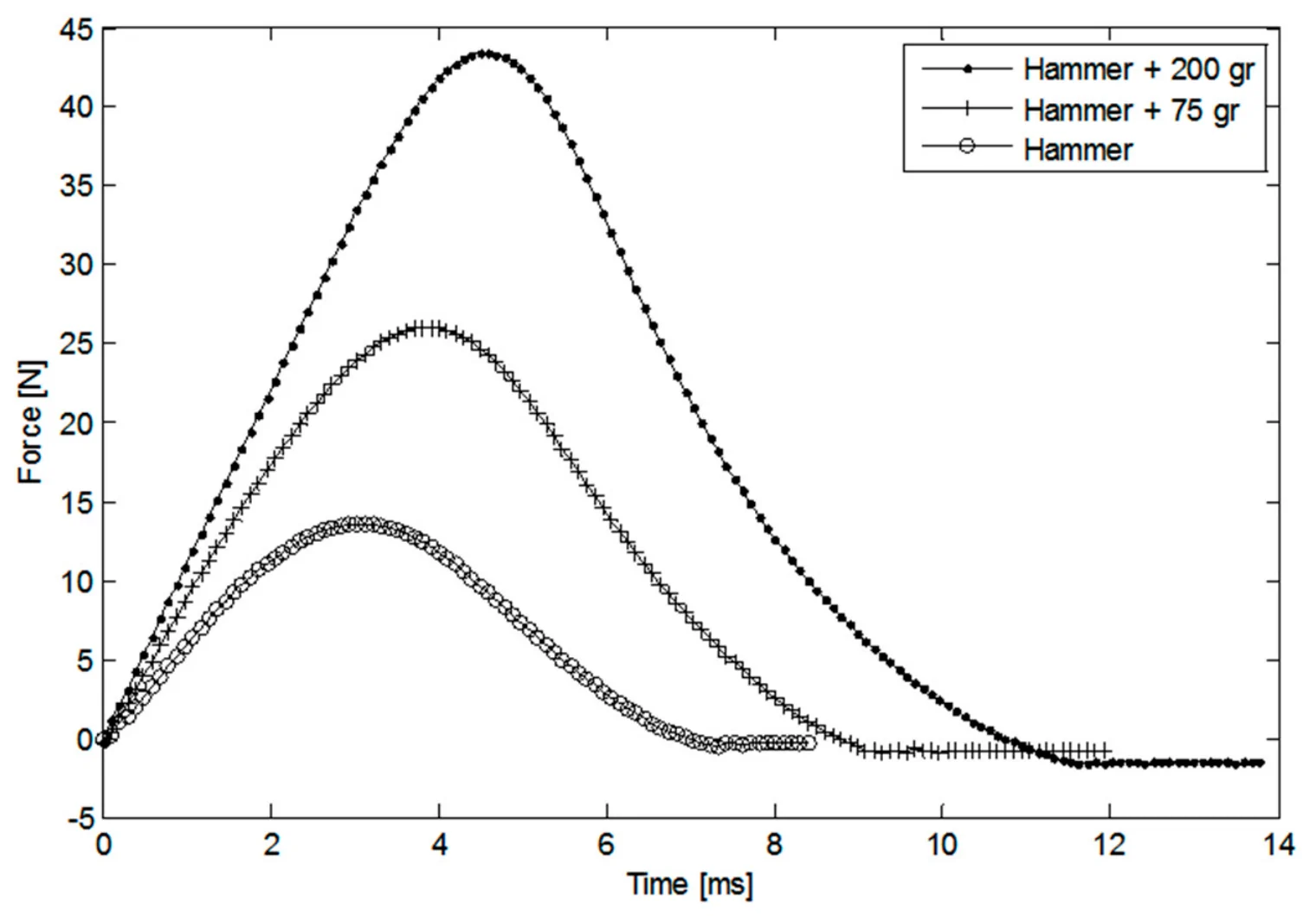
Impact force-time histories for the different hammer masses considered in the tests.

To investigate the effect of hammer mass on the beam response, additional masses were attached to the hammer, resulting in total masses of 325, 400, and 575 g. The same soft impactor head and initial velocity were used in all tests. Figure 7 shows the experimental force-time histories. As expected, the impulse magnitude increased with hammer mass. Both the peak force and the contact duration also increased.

### 4.4. Numerical Simulations

Under classical pendulum-impact theory, all cases analysed in this study have the same initial impact velocity: $v_{0}=\sqrt{2gh}$ (see Figure 1). This simplification implies that the beam response is independent of the impactor-head stiffness; i.e., the response only depends on the initial velocity and the hammer mass.

To obtain a more realistic prediction, the numerical simulations were driven by the experimental force-time histories measured with the hammer load cell.

A modal-dynamic analysis was performed in ABAQUS using the first five beam modes. The experimental damping ratios identified by OMA were assigned to the corresponding modes, and the beam was excited by the measured force-time histories.

Compliant impactors generally remain in contact with the specimen for longer and introduce less high-frequency force content than stiff impactors, promoting the excitation of lower structural modes. This provides a physical rationale for using a reduced modal representation in soft-impact applications. Here, the first five identified modes were retained rather than adopting a single-mode approximation. Their use was assessed through comparison with the measured responses, although a modal-truncation convergence study was not performed.

**Figure 8:**
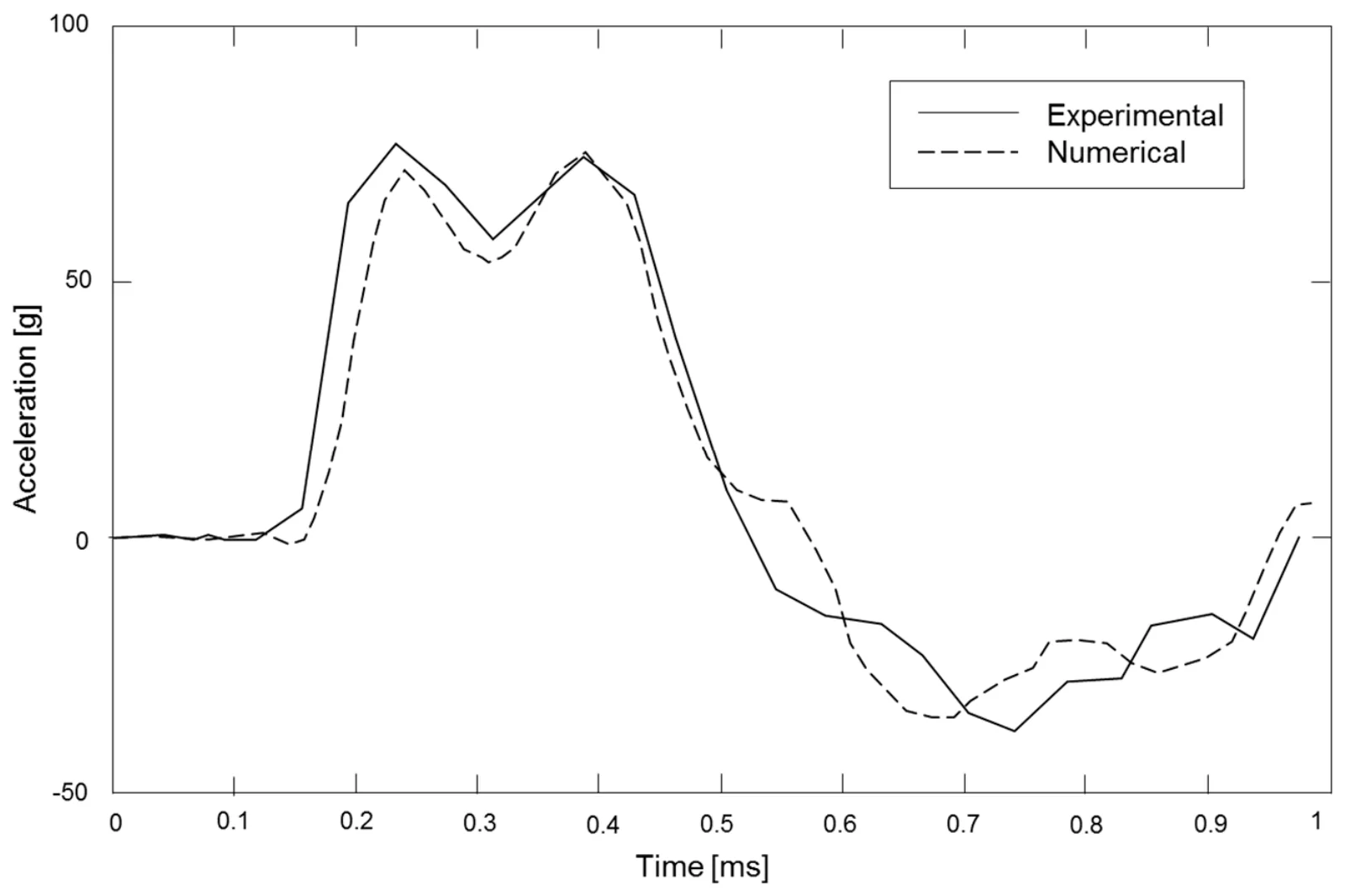
Experimental and numerical acceleration at the beam midpoint for the hard impactor head.

Figure 8 compares the acceleration measured at the beam midpoint with that predicted by the numerical model for the hard impactor head. The measured and numerical responses agree well during the coupling interval. The remaining differences are consistent with the discrepancies between the experimental and numerical natural frequencies. Comparable trends were obtained for the other impactor heads.

For the acceleration comparison in Figure 8, digitisation of the plotted curves gives an approximate normalised root-mean-square error (NRMSE) of 12.1%, an absolute-peak error of 2.1% and a Pearson correlation coefficient of 0.972 over their common plotted interval. The NRMSE and absolute-peak error are referenced to the maximum absolute experimental acceleration.

These results show that, although direct simulation of the impactor-beam coupling is difficult, a simplified linear modal analysis can provide accurate response predictions when it is driven by the measured force-time history.

The reported configurations illustrate the application of the method; repeated-test variability was not quantified. Changes in release height are reflected in the measured response, and the formulation can be applied to other conditions provided that the retained modal basis, processing, and material description remain appropriate. Temperature changes also require representative modal information and stress-effective moduli at the corresponding temperature. This physical adaptability does not establish repeatability or accuracy under untested conditions.

## 5. Laminated Glass Beam

### 5.1. Modal Analysis

**Figure 9:**
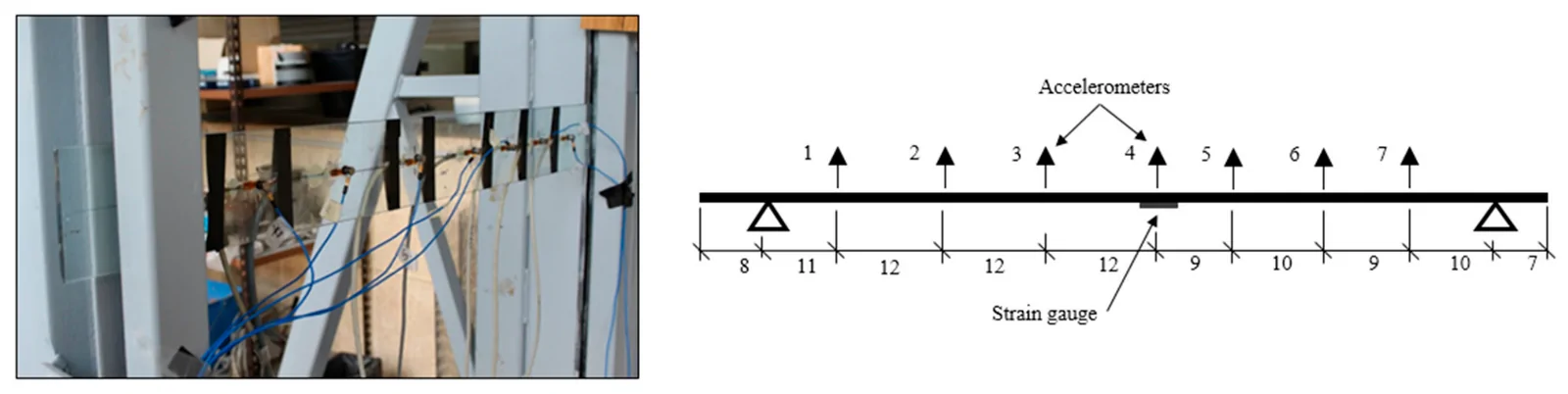
Experimental setup for the laminated glass beam (distances in cm).

A laminated glass beam with the geometrical properties listed in Table 3 was tested. The beam was clamped at both ends between rubber pads in the standard glass impact frame [7] shown in Figure 1; the sensor layout is presented in Figure 9.

**Figure 10:**
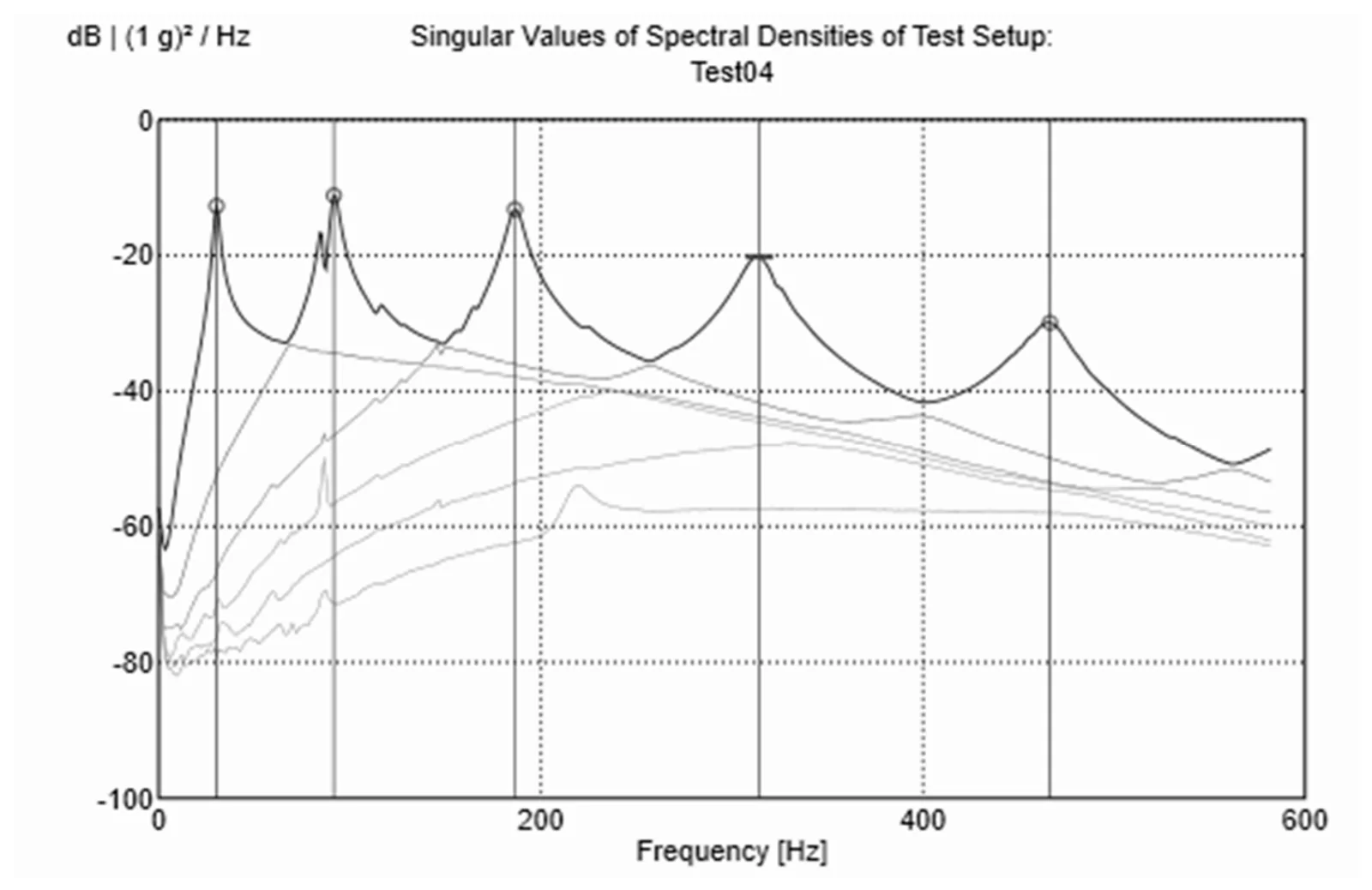
Singular-value decomposition of the response spectral-density matrix for the laminated-beam OMA test.

The modal parameters of the laminated beam were identified by OMA at 22 °C. Seven accelerometers with a sensitivity of 10 mV/g were uniformly distributed along the beam. Seven 350 Ω unidirectional HBM strain gauges, oriented longitudinally along the beam, were installed at the locations shown in Figure 9. Accelerometer, strain-gauge, and hammer-load-cell signals were acquired using a National Instruments CompactDAQ system at a sampling frequency of 2132 Hz for approximately 2 min. The OMA records were processed in ARTeMIS Modal, and the modal parameters were estimated in the frequency domain using the same EFDD procedure as for the monolithic beam. Figure 10 presents the singular-value decomposition of the response spectral-density matrix, and Table 4 lists the first five natural frequencies, damping ratios, and mode shapes.

The seven approximately equally spaced accelerometers were selected to provide spatial coverage for identifying the first five modes and were also used for the impact measurements. The layout was intended to capture the spatial variation of the target mode shapes, rather than to establish the minimum sensor count required for stress reconstruction. For the five-mode reconstruction used here, the additional measurement channels provide an overdetermined system, although its conditioning also depends on sensor position and the mode-shape amplitudes at the measured locations.

### 5.2. Impact Test

The laminated beam was subjected to an impact test using the medium-stiffness head and an additional hammer mass of 75 g. The beam response was measured using the sensor configuration employed in the modal test (Figure 9).

### 5.3. Sensor-Based Analytical Predictions

The modal coordinates were estimated from the experimental responses and mode shapes using Equation (12). The experimental mode shapes were then expanded using Equations (15) and (16). A monolithic shell finite element model was assembled in ABAQUS using the glass properties given in Section 4.1 and 870 linear quadrilateral S4R elements. A mesh-sensitivity study was used to check the adequacy of the adopted discretisation. The supports were represented as pinned, an idealisation retained on the basis of agreement with the experimental modal results. The transformation matrix T (Table 5), calculated using Equation (16), combines the numerical mode shapes to expand the experimental modes and obtain the modal curvatures used in stress reconstruction.

The agreement between the experimental and numerical mode shapes was quantified using the modal assurance criterion (MAC), evaluated at the measured degrees of freedom. The diagonal MAC values for modes 1–5 were 0.9996, 0.9992, 0.9991, 0.9966, and 0.9465, respectively; all off-diagonal values were at most 0.0622. These results support the use of the numerical mode shapes as a spatial basis for expansion. In this reconstruction, the modal coordinates are obtained from the measured response, which contains the actual frequency and damping characteristics; the numerical natural frequencies do not prescribe their time evolution. Consequently, differences between numerical and experimental frequencies do not directly determine the reconstructed response. The accuracy of the spatial mode shapes and their curvatures remains important, and MAC agreement at the measured locations does not independently validate curvatures at unmeasured locations.

**Figure 11:**
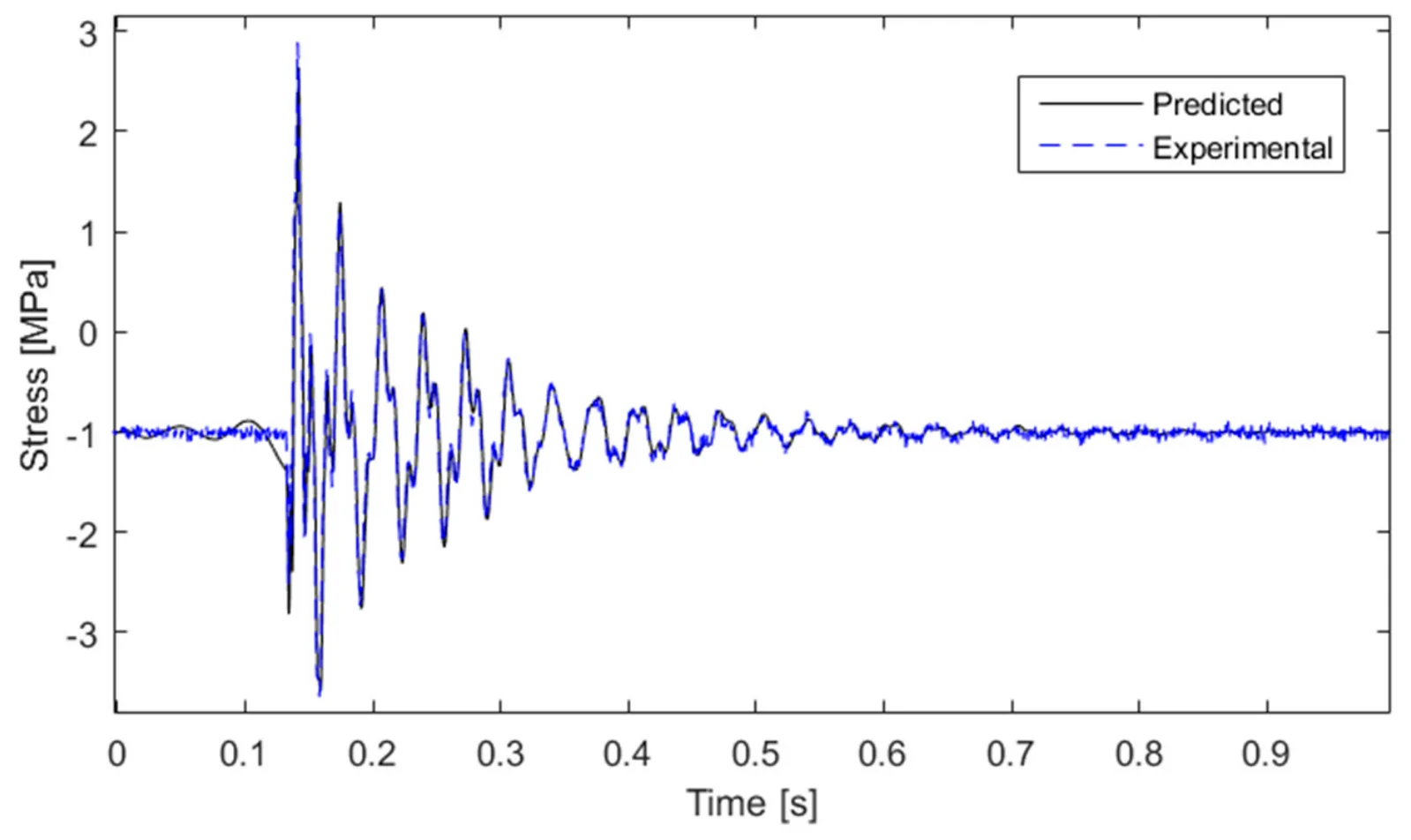
Predicted and measured stress at the midpoint of the laminated glass beam under soft impact loading.

The stress predicted at the midpoint of the bottom surface of glass layer H_3_ using Equation (14) is compared in Figure 11 with the stress obtained from the strain gauge at the same location. The effective Young’s modulus at T = 22 °C was calculated using Equation (A2).

For the stress comparison in Figure 11, digitisation of the plotted curves gives an approximate NRMSE of 3.8%, an absolute-peak error of 1.5% and a Pearson correlation coefficient of 0.943 over their common plotted interval. The NRMSE and absolute-peak error are referenced to the maximum absolute measured stress. These metrics characterise agreement for the tested configuration.

The midpoint was selected for the comparison as a location of maximum bending stress for the tested configuration. Once the modal coordinates are available, Equations (13) and (14) can be evaluated at other longitudinal positions using the corresponding expanded modal curvatures. The reconstruction is therefore not restricted to the strain-gauge location. To keep the presentation focused, comparisons at the other instrumented locations are not included; the results shown do not quantify spatial reconstruction accuracy at those locations.

The test at 22 °C provides an illustrative assessment in the room-temperature region where PVB exhibits pronounced viscoelastic sensitivity. Bennison et al. [4] report rapid softening and a glass transition around room temperature for PVB. This makes the present condition relevant for assessing the reconstruction when interlayer properties depend strongly on temperature and loading time scale.

According to time-temperature superposition [1], temperature changes can be represented by horizontal shifts along the logarithmic time or frequency axis, within the range where this principle applies. At sufficiently short reduced times, the interlayer modulus approaches its glassy limit E_0_. Short-duration impacts therefore favour a comparatively stiff interlayer response, although the impact duration alone does not establish that all contributing modal frequencies lie on the glassy plateau at every temperature.

Moreover, the temperature-induced changes in structural stiffness and damping are reflected in the measured accelerations. Thus, application at another temperature follows the same formulation, with consistent material properties and modal information, including the mode-shape expansion model. The physical basis above supports applicability beyond the temperature of this example, while the quantitative agreement reported here remains that measured at 22 °C. The comparison with experimental measurements supports the potential of the approach for reconstructing stress histories under soft-impact loading. The formulation also permits stress reconstruction at other longitudinal positions through the expanded modal curvatures in Equations (13) and (14). Application to other configurations requires representative modal information and material properties consistent with the underlying modelling assumptions. The comparison presented here illustrates this capability for the tested configuration.

A smaller sensor set could be sufficient when fewer modes adequately describe the response of interest, as may occur when a soft impact predominantly excites the first bending mode. However, this possibility requires verification for stress reconstruction, since higher modes can contribute appreciably to curvature even when their displacement contribution is small. The numerical mode shapes provide the spatial basis for expansion, while the experimental measurements determine the transformation matrix T. With insufficient independent measurements, this estimation becomes underdetermined and requires a reduced modal basis or additional modelling assumptions. No sensor-number or sensor-location sensitivity study was performed here. Future work will assess these effects and investigate sensor layouts selected specifically for curvature and stress estimation.

## 6. Conclusions

When the dynamic response of glass panels under impact loading is predicted using simplified analytical or monolithic models, energy-balance methods are commonly used to determine the initial impact velocity from the release height. For soft impacts, however, the response is influenced by the transient coupling between the specimen and the impactor, including the impactor-head stiffness and hammer mass. This coupling should therefore be considered to obtain realistic predictions.

The dynamic response of monolithic and laminated glass beams was investigated experimentally using an impactor with different heads and additional masses. The tests confirmed the existence of transient coupling when soft impactor heads were used. The coupling duration varied with impactor stiffness and mass.

The coupling effect may be represented by a finite element model that includes both the glass element and the impactor. Such models are, however, computationally demanding and difficult to implement. For laminated glass, the viscoelastic behaviour of the interlayer further increases the modelling complexity.

A simplified method was therefore applied and experimentally assessed in which only the glass element is modelled, and the measured impact-force history is applied. The method combines experimental modal parameters with the force-time history, which may be measured directly or estimated from the impactor mass and the acceleration recorded by a sensor mounted on the impactor. For the monolithic beam, the digitised acceleration comparison gives an approximate NRMSE of 12.1% relative to the maximum absolute measured acceleration, with a Pearson correlation coefficient of 0.972.

A second approach estimates stress using only the measured structural responses, without requiring the impact-force history. For laminated glass, the formulation incorporates a time- and temperature-dependent stress-effective Young’s modulus, whose analytical expressions are provided in Appendix A. For the tested laminated beam, the digitised stress comparison gives an approximate NRMSE of 3.8% relative to the maximum absolute measured stress, with a Pearson correlation coefficient of 0.943. The sensor-based reconstruction approach may support structural assessment and fatigue-oriented structural health monitoring by providing stress histories at locations where direct strain instrumentation is unavailable. In the present study, however, the method was validated only for short-duration soft-impact tests on beam specimens; damage detection and fatigue-life prediction were outside the scope of the work.

The test at 22 °C illustrates the method in the temperature-sensitive region of PVB behaviour. Temperature effects can be incorporated through measured structural responses, modal information representative of the operating condition, and stress-effective moduli evaluated at the corresponding temperature and modal frequencies. This provides a physical basis for application at other temperatures using the same formulation. The reported experimental agreement concerns one laminated beam and one stress-comparison location; repeatability and spatial accuracy were not quantified.

## Figures and Tables

**Table 1:** Dimensions and mass of the monolithic beam.

Length [mm]	Width [mm]	Thickness [mm]	Mass [kg]
1000	100	9	2.248

**Table 2:** Experimental modal parameters and comparison with numerical natural frequencies and mode shapes for the monolithic glass beam.

Mode	Natural Frequencies [Hz]	Exp. Damp. [%]	Num. Freq. [Hz]	Error Freq. [%]	Mode Shape
1	31.013	2.637	28.471	8.01	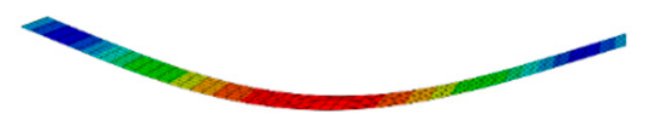
2	106.76	1.332	113.37	6.19	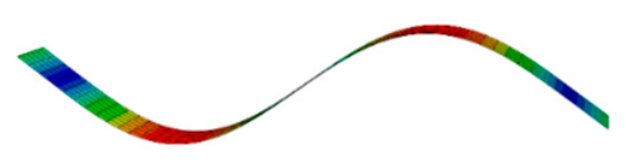
3	230.39	0.997	252.95	9.78	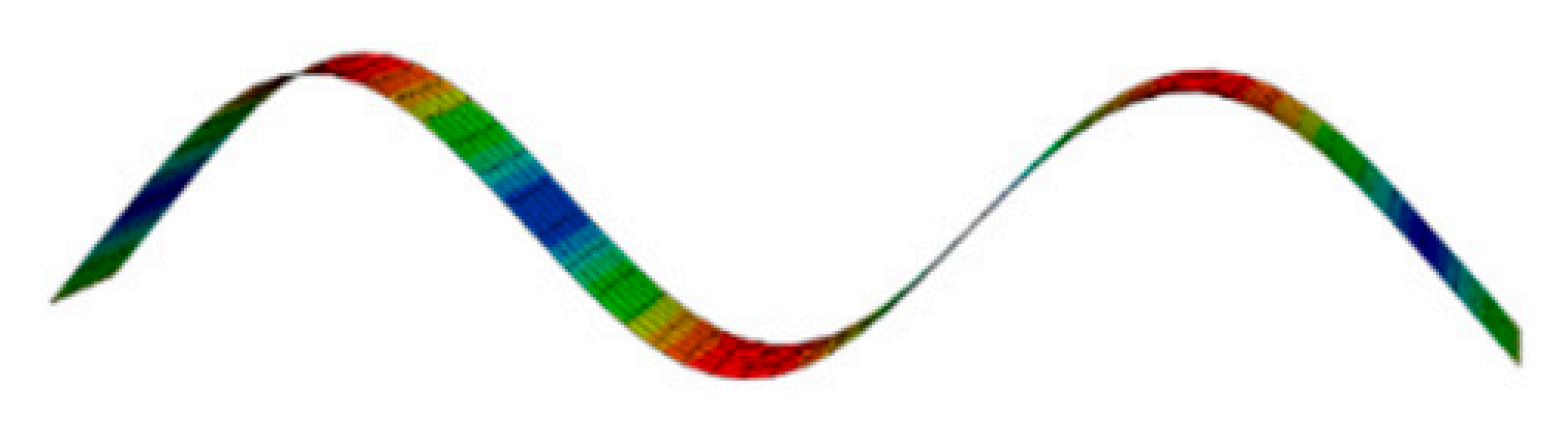
4	397.70	0.5031	421.20	6.09	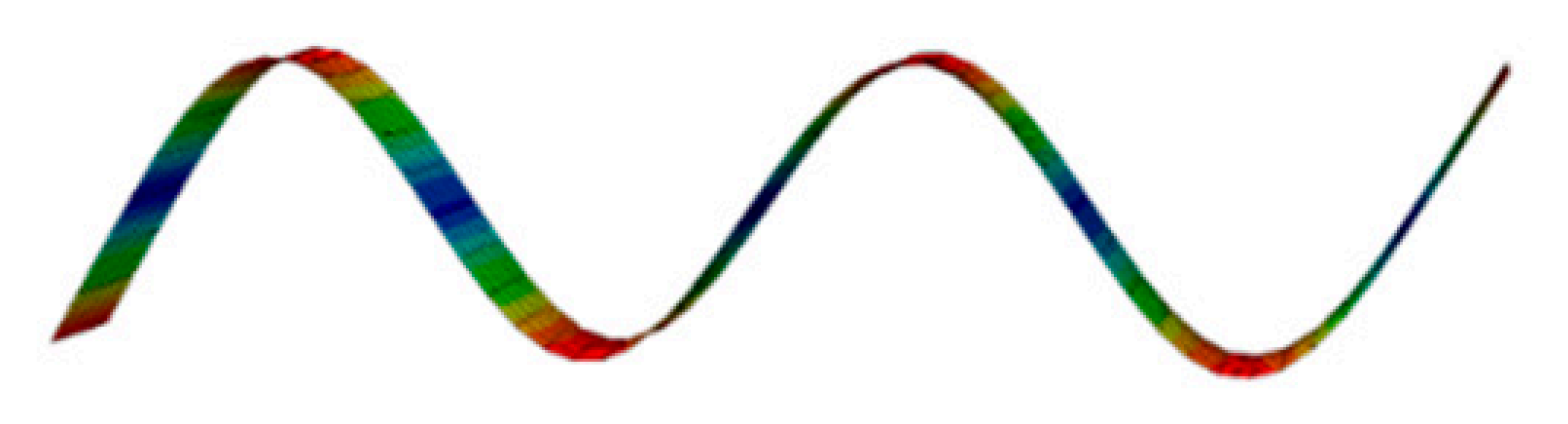
5	599.61	0.4371	649.87	8.34	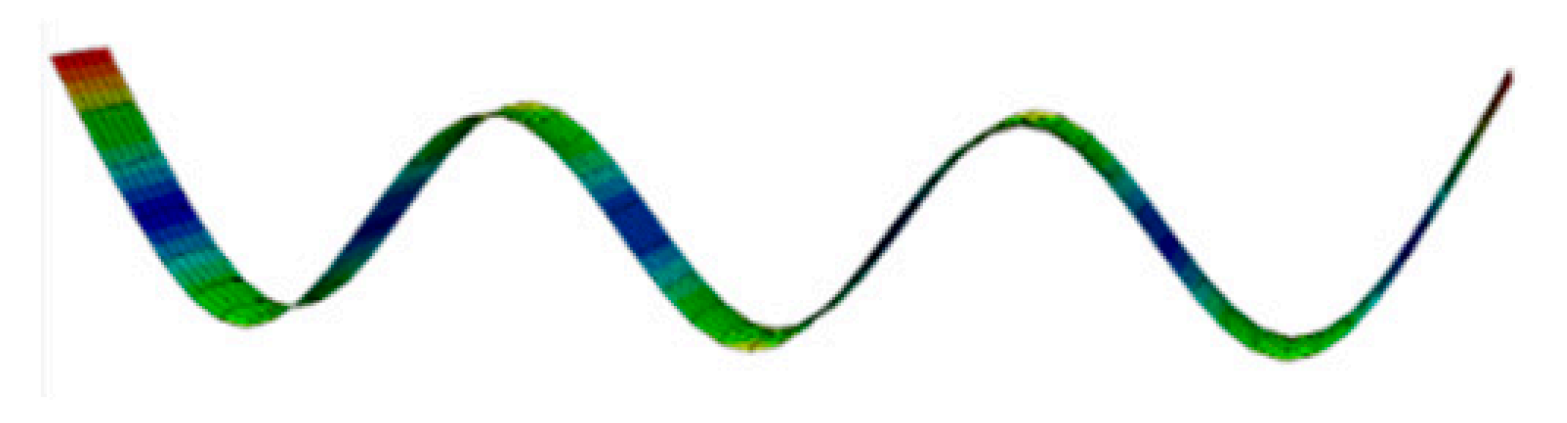

**Table 3:** Dimensions and mass of the laminated glass beam.

Length [mm]	Width [mm]	H_1_ [mm]	H_2_ [mm]	H_3_ [mm]	Mass [kg]
1000	100	3	0.38	3	1.540

**Table 4:** Experimental modal parameters of the laminated glass beam.

Mode	Natural Frequencies [Hz]	Exp. Damp. [%]	Experimental Mode Shape
1	30.16	4.28	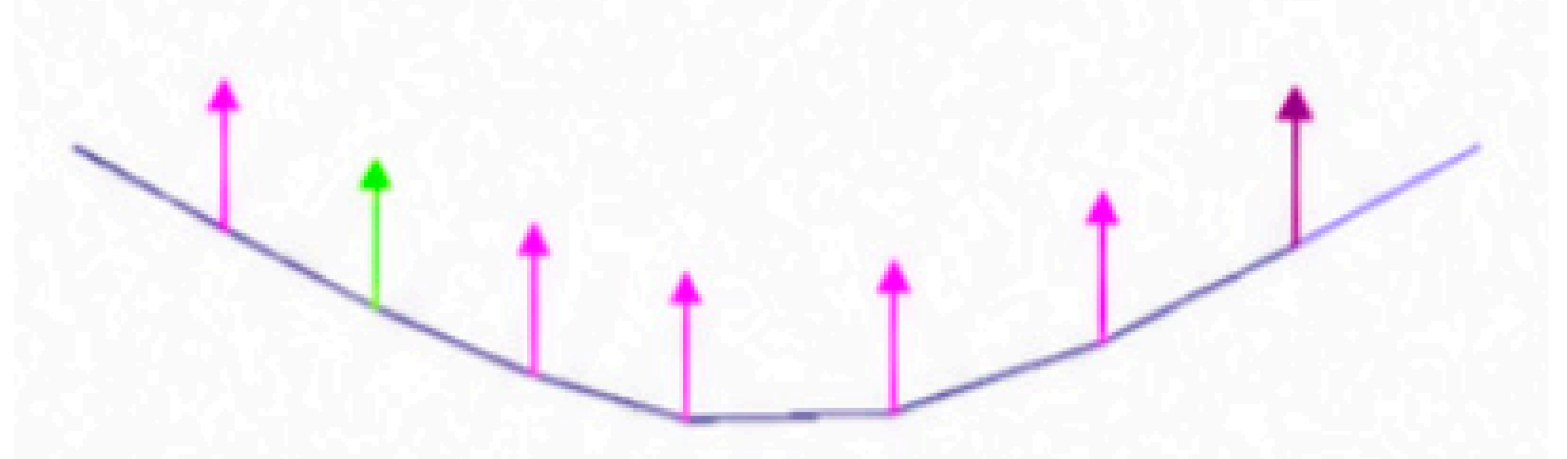
2	92.70	3.23	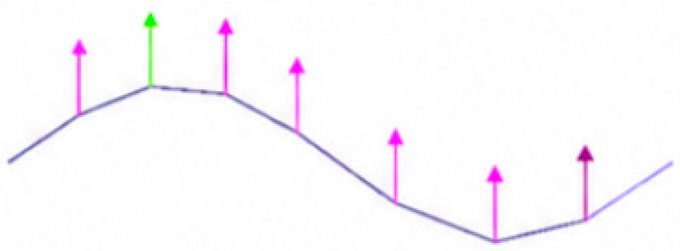
3	186.49	2.92	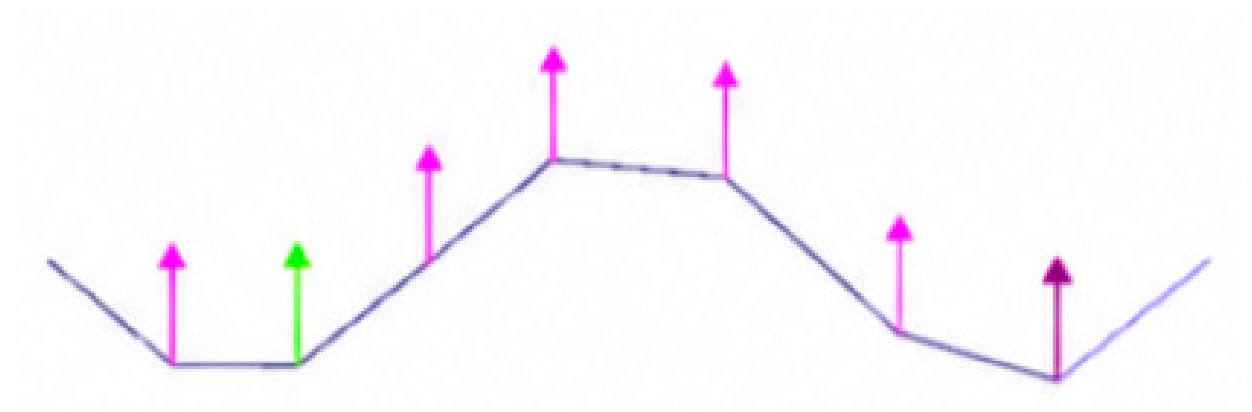
4	313.41	2.89	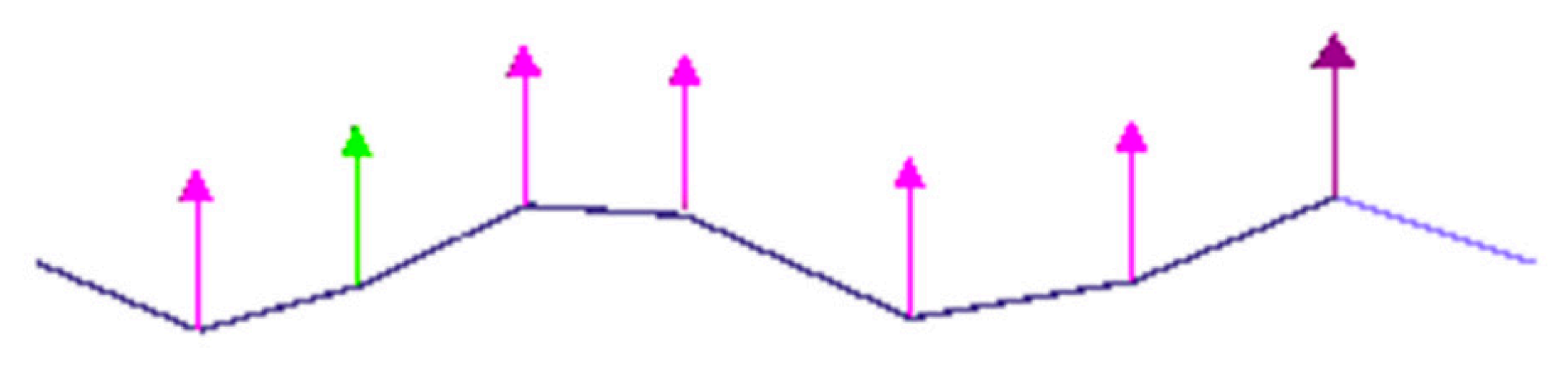
5	465.53	2.82	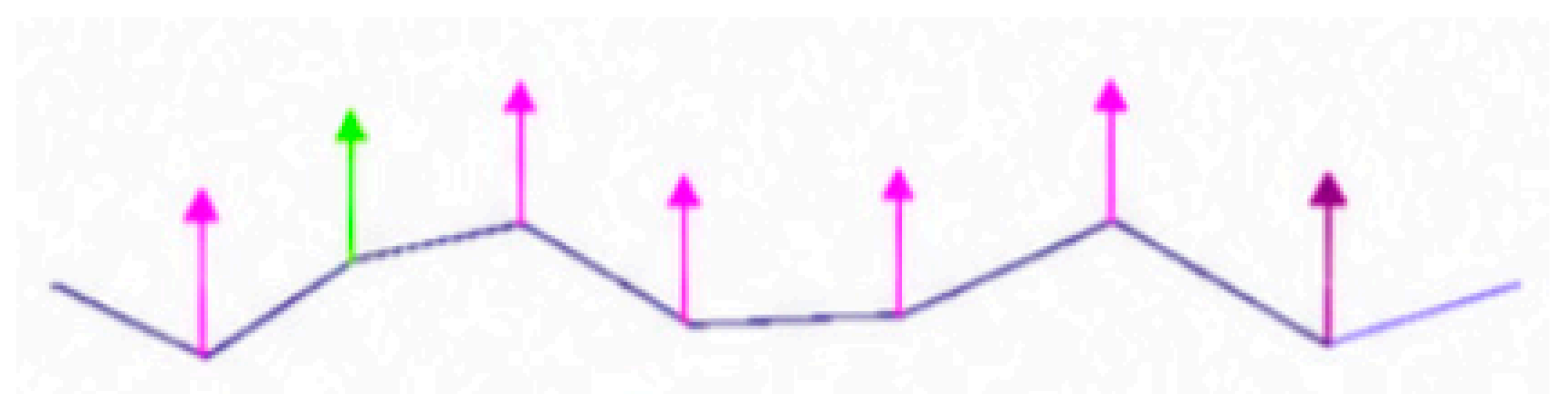

**Table 5:** Transformation matrix *T*.

0.9952	−0.0005	0.0070	−0.0201	−0.0638
0.0130	0.9967	0.0217	−0.0411	−0.0476
0.0035	0.0254	0.9642	−0.0148	−0.1527
−0.0069	−0.0142	−0.0069	−0.0194	0.9863
−0.0132	−0.0080	−0.0043	−0.0145	1.1590

**Figure A1:**
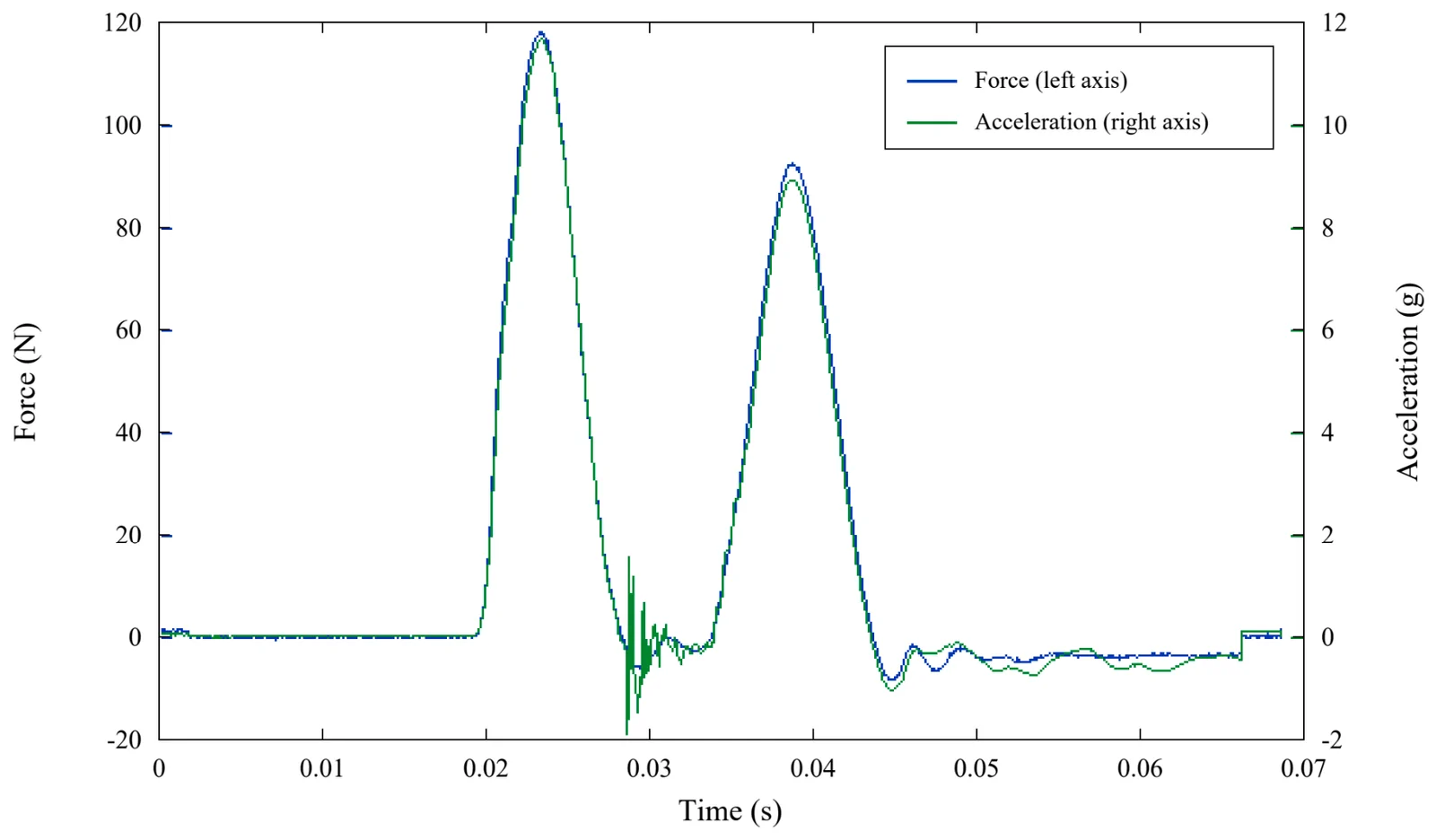
Measured hammer force and acceleration for the super-soft tip.

**Figure A2:**
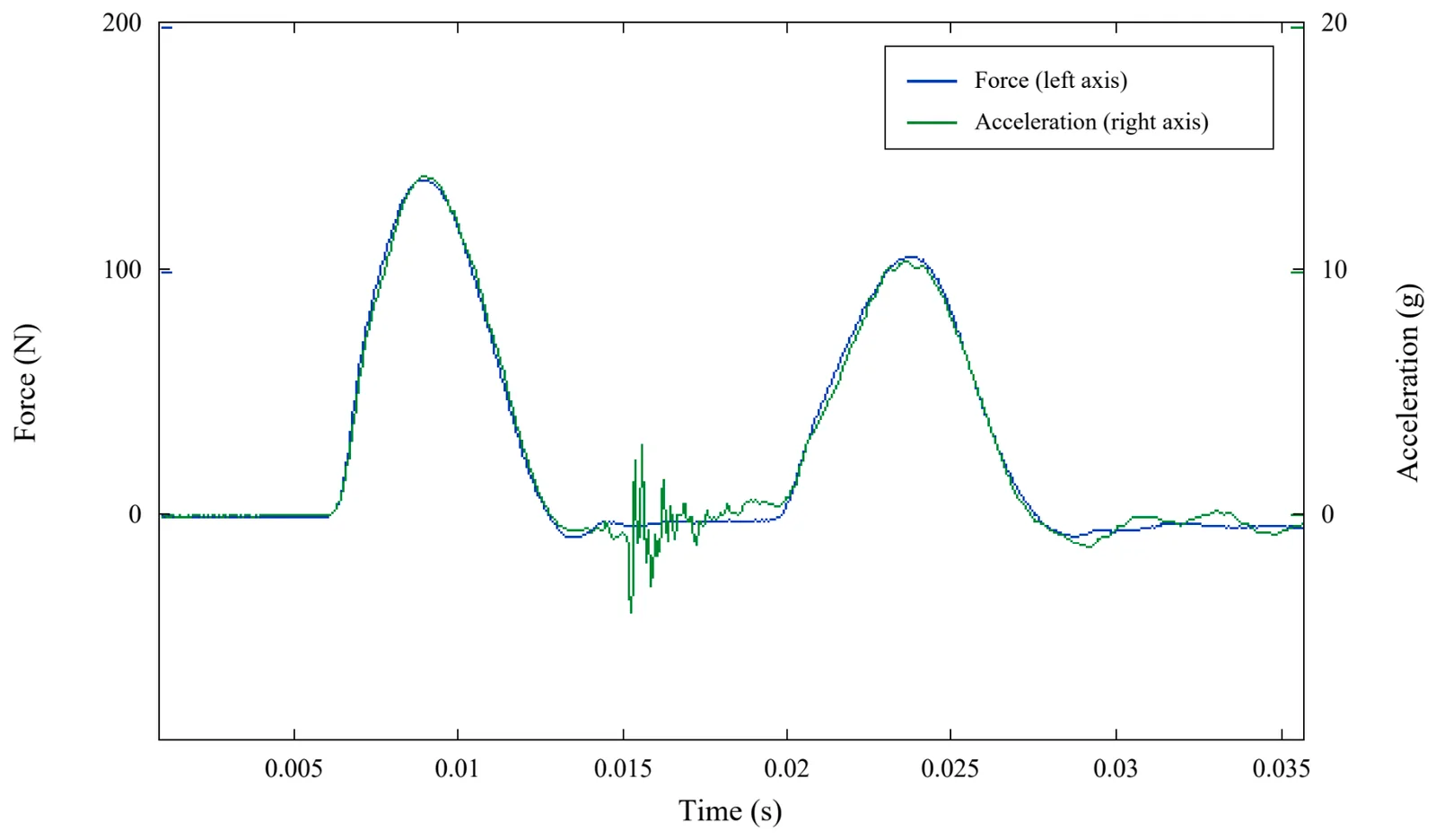
Measured hammer force and acceleration for the soft tip.

## Data Availability

The data supporting the findings of this study are available from the corresponding author upon reasonable request.

## Appendix A

The expressions below use equal glass-ply moduli, $E_{1}\;=\;E_{3}\;=\;E$, and section properties per unit width. The fully coupled glass-section inertia is $I_{TOT}\;=\;I_{T}(1\;+\;Y)$. Here, $H_{2}$ is the interlayer thickness and $k_{I}$ is the beam wavenumber; its square appears in Equations (A3) and (A4).

$$H_{0}=H_{2}+\left(\frac{H_{1}+H_{3}}{2}\right)$$

$$Y=\frac{H_{0}^{2}E_{1}H_{1}{E_{3}H}_{3}}{{EI}_{T}(E_{1}H_{1}{{+E}_{3}H}_{3})}$$

$$I_{T}=I_{1}+I_{3}=\frac{H_{1}^{3}+H_{3}^{3}}{12}$$

$$I_{1}=\frac{H_{1}^{3}}{12}$$

$$I_{3}=\frac{H_{3}^{3}}{12}$$

$E_{1}$: Young’s modulus of glass layer 1
$E_{3}$: Young’s modulus of glass layer 3
$G_{2}^{*}(\omega )$: Complex shear modulus for the polymeric interlayer
$k_{I}^{2}\left(\omega ,T\right)$: Squared wavenumber of the beam

(A1) $$E_{1\sigma eff}\left(\omega ,T\right)=E\left[\eta_{d}\left(\omega ,T\right)\cdot \frac{EI^{*}\left(\omega ,T\right)}{EI_{TOT}}\frac{H_{3}H_{0}}{H_{1}+H_{3}}\cdot \frac{2}{H_{1}}+1\right]$$

(A2) $$E_{3\sigma eff}\left(\omega ,T\right)=E\left[\eta_{d}\left(\omega ,T\right)\cdot \frac{EI^{*}\left(\omega ,T\right)}{EI_{TOT}}\frac{H_{1}H_{0}}{H_{1}+H_{3}}\cdot \frac{2}{H_{3}}+1\right]$$

(A3) $$EI^{*}(\omega ,T)=EI_{T}\;\left(1+\frac{Y}{1+\frac{{{H_{2}E}_{1}H}_{1}E_{3}H_{3}k_{I}^{2}\left(\omega ,T\right)}{G_{2}^{*}\left(\omega ,T\right)({E_{1}H}_{1}+{E_{3}H}_{3})}}\right)$$

(A4) $$\eta_{d}(\omega )=\frac{1}{1+\frac{E_{1}\;H_{1}\;H_{2}E_{3}\;H_{3}\;{k_{I}}^{2}}{G_{2}^{*}\;(\omega )\left({E_{1}H}_{1}+{E_{3}H}_{3}\right)(1+Y)}}$$

## Appendix B. Hammer Force and Acceleration Comparison

Figure A1 and Figure A2 compare the measured force and hammer acceleration for the super-soft and soft tips. The hammer is approximated as a rigid pendulum with its mass concentrated at the centre of gravity. The total mass is 1.18 kg, the pivot-to-centre-of-gravity distance is 0.32 m, and the impact point and accelerometer are 0.34 m from the pivot. Neglecting the distributed inertia about the centre of gravity gives $I_{O}\approx m{r_{G}}^{2}$. Equating rotational kinetic energy to that of a mass moving at the impact-point velocity gives [38]:

(A5) $$\frac{1}{2}I_{O}\omega^{2}=\frac{1}{2}m_{eff}{v_{P}}^{2},\;\;\;\;v_{P}=r_{P}\omega$$

For tangential contact near the bottom of the swing, with gravitational and pivot-friction torques neglected during the short contact interval, this equivalent mass relates the contact-force magnitude to the tangential deceleration at the impact point. Thus,

(A6) $$m_{eff}=\frac{I_{O}}{{r_{P}}^{2}}\approx 1.18{\left(\frac{0.32}{0.34}\right)}^{2}\approx 1.05\;kg$$
